# The *OsOXO2*, *OsOXO3* and *OsOXO4* Positively Regulate Panicle Blast Resistance in Rice

**DOI:** 10.1186/s12284-021-00494-9

**Published:** 2021-06-05

**Authors:** Jingfang Dong, Lian Zhou, Aiqing Feng, Shaohong Zhang, Hua Fu, Luo Chen, Junliang Zhao, Tifeng Yang, Wu Yang, Yamei Ma, Jian Wang, Xiaoyuan Zhu, Qing Liu, Bin Liu

**Affiliations:** 1 Rice Research Institute, Guangdong Academy of Agricultural Sciences, Guangzhou, 510640 China; 2 Guangdong Key Laboratory of New Technology in Rice Breeding, Guangzhou, 510640 China; 3 Guangdong Key Laboratory of New Technology in Plant Protection, Plant Protection Research Institute, Guangdong Academy of Agricultural Sciences, Guangzhou, 510640 China

**Keywords:** Rice (*Oryza sativa* L.), Panicle blast, OXO (oxalate oxidase)

## Abstract

**Background:**

Although panicle blast is more destructive to yield loss than leaf blast in rice, the cloned genes that function in panicle blast resistance are still very limited and the molecular mechanisms underlying panicle blast resistance remain largely unknown.

**Results:**

In the present study, we have confirmed that the three Oxalate oxidase (*OXO*) genes, *OsOXO2*, *OsOXO3* and *OsOXO4* from a blast-resistant cultivar BC10 function in panicle blast resistance in rice. The expression of *OsOXO2*, *OsOXO3* and *OsOXO4* were induced by panicle blast inoculation. Subcellular localization analysis revealed that the three OXO proteins are all localized in the nucleus and cytoplasm. Simultaneous silencing of *OsOXO2*, *OsOXO3* and *OsOXO4* decreased rice resistance to panicle blast, whereas the *OsOXO2*, *OsOXO3* and *OsOXO4* overexpression rice plants individually showed enhanced panicle blast resistance. More H_2_O_2_ and higher expression levels of *PR* genes were observed in the overexpressing plants than in the control plants, while the silencing plants exhibited less H_2_O_2_ and lower expression levels of *PR* genes compared to the control plants. Moreover, phytohormone treatment and the phytohormone signaling related gene expression analysis showed that panicle blast resistance mediated by the three *OXO* genes was associated with the activation of JA and ABA signaling pathways but suppression of SA signaling pathway.

**Conclusion:**

*OsOXO2*, *OsOXO3* and *OsOXO4* positively regulate panicle blast resistance in rice. The *OXO* genes could modulate the accumulation of H_2_O_2_ and expression levels of *PR* gene in plants. Moreover, the *OXO* genes mediated panicle blast resistance could be regulated by ABA, SA and JA, and may be associated with the activation of JA and ABA signaling pathways but suppression of the SA signaling pathway.

**Supplementary Information:**

The online version contains supplementary material available at 10.1186/s12284-021-00494-9.

## Background

Rice (*Oryza sativa* L.) is a major food crop for more than half of the world’s population. The demand is increasing with growing population. However, rice production is facing many challenges. Rice blast disease, caused by *Magnaporthe oryzae* (*M. oryzae*) is one of the most destructive diseases in rice plant, causing 10% - 30% of yield loss every year (Skamnioti and Gurr 2009). Use of resistant variety is considered to be the most economical and environment-friendly approach to solve this problem (Hu et al. 2008). Rice blast can be classified into leaf blast and panicle blast based on the infected parts, and generally panicle blast is more destructive in terms of yield loss (Sirithunya et al. 2002; Zhuang et al. 2002; Liu et al. 2016a; Liu et al. 2016b). Recently, more and more studies have shown that the correlations between leaf blast and panicle blast are not always positive, and there are different regulation mechanisms between leaf blast resistance and panicle blast resistance (Zhuang et al. 2002; Liu et al. 2016b; Fang et al. 2019). But, nowadays, almost all the work in rice blast is focused on leaf blast, and few genes for panicle blast resistance have been cloned (Liu et al. 2016a; Liu et al. 2016b; Inoue et al. 2017). Besides, our knowledge on the mechanism of panicle blast resistance is still very limited. Since blast disease may occur in different developmental stages and the blast pathogen may infect different parts (leaf or panicle) in rice, it is necessary for effective disease control to identify the genes associated with panicle blast resistance and understand their regulatory mechanisms.

Oxalate oxidases (OXOs) which belong to the germin protein family have been demonstrated to play important roles in various environmental stresses in plants (Hu et al. 2003; Dong et al. 2008; Wan et al. 2009; Partridge-Telenko et al. 2011; Karmakar et al. 2016). OXO catalyzes the degradation of oxalic acid to produce hydrogen peroxide (H_2_O_2_) and carbon dioxide. H_2_O_2_ generated from the reaction can function as a secondary messenger to activate the hypersensitive response, the phytoalexin biosynthetic pathways as well as the expression of pathogenesis-related (PR) genes in plants (Hammond-Kosack et al. 1994; Lamb and Dixon 1997; Carter et al. 1998). Owing to the function of H_2_O_2_, *OXO* genes were speculated to play a key role in plant disease resistance. Indeed, many studies have indicated the involvement of OXOs in plant basal host resistance. For example, overexpressing a wheat *OXO* gene (*gf*-2.8) resulted in the induction of defense proteins and increased resistance to *Sclerotinia sclerotiorum* in sunflower (Hu et al. 2003). Transgenic oilseed rape plants overexpressing a wheat *OXO* gene (*pSBGer2*) exhibited enhanced resistance to *Sclerotinia sclerotiorum* (Dong et al. 2008). Recently, Yang et al. (2019) reported that overexpressing a wheat *OXO* gene (GenBank No M21962.1) also showed enhanced resistance to *Sclerotinia stem rot* in *Glycine max* (Yang et al. 2019).

In rice plants, four *OXO* genes (*OsOXO1*–*OsOXO4*) with > 90% nucleotide sequence identity are identified on chromosome 3. They form a tandemly duplicated cluster and co-localize with a blast disease resistance QTL (Ramalingam et al. 2003; Wu et al. 2004). Existing studies have shown that *OsOXO4* was expressed during rice-*M. oryzae* infection in leaf blast, and the expression of *OsOXO4* increased earlier in blast-resistant cultivar Moroberekan than blast-susceptible cultivar Vandana (Carrillo et al. 2009). Moreover, overexpressing *OsOXO4* driven by the green tissue-specific promoter and co-expression of *OsCHI11* and *OsOXO4* all showed increased resistance to sheath blight pathogen in rice (Molla et al. 2013; Karmakar et al. 2016). Although the four *OXO* genes (*OsOXO1*–*OsOXO4*) have been demonstrated to co-localize with a blast resistant QTL, their specific regulation roles in rice blast resistance have not been reported so far.

In the present study, we identified that the transcription levels of *OsOXO2*, *OsOXO3* and *OsOXO4* were significantly induced by panicle blast inoculation. To confirm their functions in panicle blast resistance, we performed sub-cellular localization, spatio-temporal expression and transgenic analysis. Our results showed that *OsOXO2*, *OsOXO3* and *OsOXO4* cloned from a blast-resistant line BC10 function as positive regulators of panicle blast resistance in rice. The *OXO* genes could modulate the accumulation of H_2_O_2_ and expression levels of *PR* gene in plants. Moreover, the *OXO* genes mediated panicle blast resistance could be regulated by ABA, SA and JA, and may be associated with the activation of JA and ABA signaling pathways but suppression of the SA signaling pathway.

## Results

### The *OXO* Genes Exhibit Different Temporal and Spatial Expression Patterns and are Localized in Nucleus and Cytoplasm in Rice

**Fig. 1:**
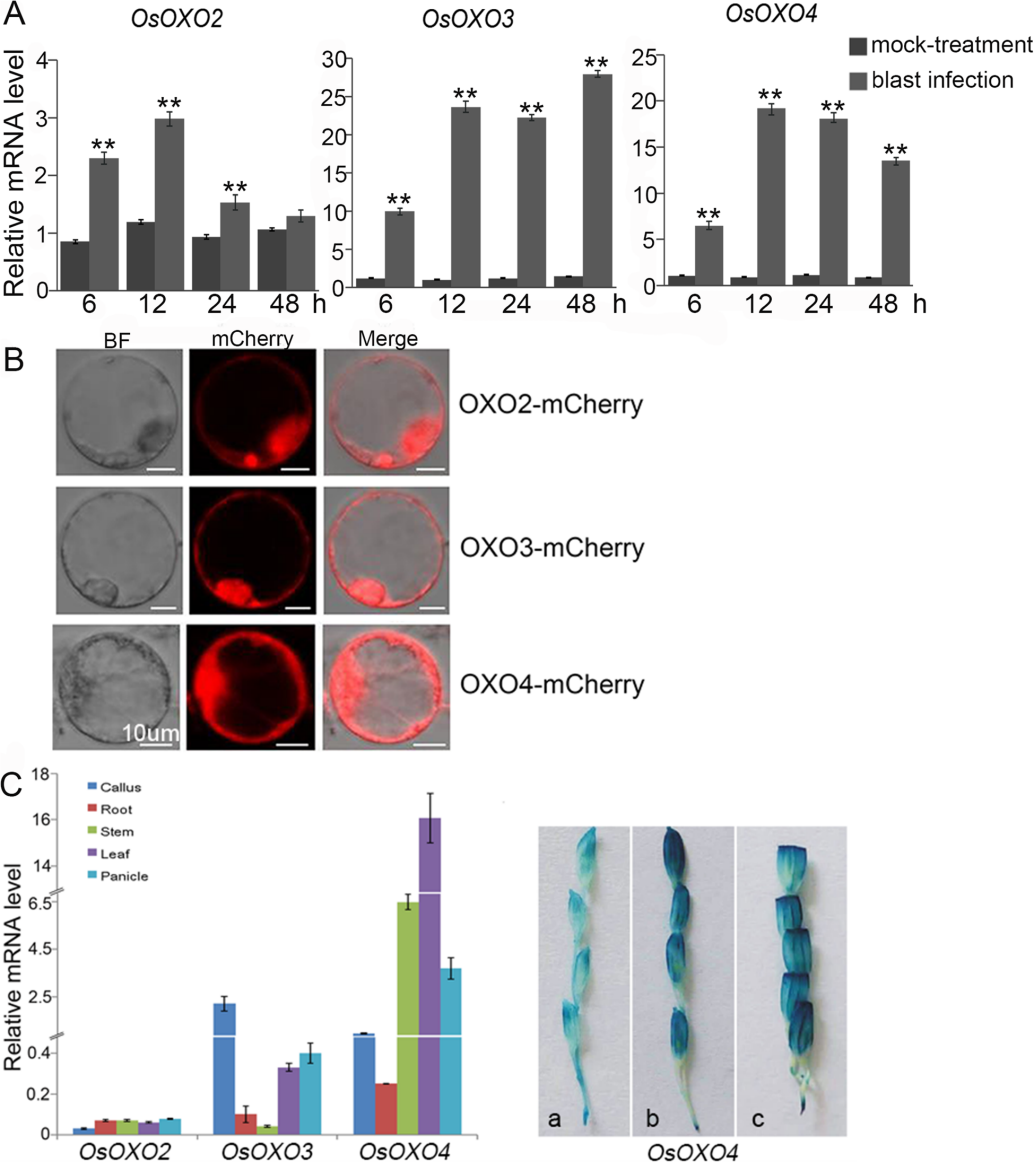
The temporal and spatial expression patterns of *OsOXO2*, *OsOXO3* and *OsOXO4* and their subcellular localizations. A. The expression of *OsOXO2*, *OsOXO3* and *OsOXO4* were assessed by quantitative RT-PCR at 6 h, 12 h, 24 h, 48 h after panicle blast inoculation. Error bars indicate the standard deviation (SD) from three biological replicates and ** indicates a statistically significant difference compared with mock (t test, *P* < 0.01). B. Sub-cellular localization of OsOXO2, OsOXO3 and OsOXO4 in rice protoplasts. Bar = 10 μm. C. Expression analysis of *OsOXO2*, *OsOXO3* and *OsOXO4* in different rice tissues by quantitative RT-PCR and GUS activity of *OsOXO4* in the panicles of *Nipponbare*. Error bars indicate the standard deviation (SD) from three biological replicates. a, panicle at the early booting stage; b, panicle at the booting stage; c, panicle at the initial heading stage

In our previous study, we have performed a microarray analysis using a blast resistant line BC10 (Liu et al. 2004) and discovered that the expression levels of the three *OXO* genes, *OsOXO2*, *OsOXO3* and *OsOXO4*, were strongly induced by panicle blast inoculation within a period of 48 h (Table S1). To further confirm these results, we analyzed the expression patterns of the three *OXO* genes in panicles in BC10 plants after blast infection using quantitative RT-PCR in this study. The results showed that the expression of *OsOXO2* in infected panicles was induced dramatically at 6 h, 12 h and 24 h after inoculation. The expression levels of *OsOXO3* also increased significantly at 6 h, 12 h, 24 h and 48 h after blast inoculation. Similarly, the transcription level of *OsOXO4* was also induced at all time points after pathogen inoculation (Fig. 1A). These results suggest the possible roles of the three *OXO* genes in regulating panicle blast resistance in rice.

To analyze the sub-cellular localization of the three *OXO* genes, we fused the coding region of them with the red fluorescent protein (mcherry) fragment under the control of the cauliflower mosaic virus 35S promoter and expressed the fusion proteins in rice protoplasts, respectively. Laser confocal microscopy showed that the red fluorescent emitted by OsOXO2-mcherry, OsOXO3-mcherry and OsOXO4-mcherry fusion proteins were located in nucleus and cytoplasm (Fig. 1B).

To investigate the temporal and spatial expression patterns of the three *OXO* genes in rice plants, we analyzed the transcription of them in various tissues of *Nipponbare* by quantitative RT-PCR using *EF1α* as the internal control. As shown in Fig. 1C, the expression of *OsOXO2* was almost hard to be detected in all rice tissues examined. *OsOXO3* expressed in callus, leaf and panicle, with the highest expression level in callus, but its background expression level is relatively low. Moreover, *OsOXO4* expressed in all rice tissues examined, with a relatively higher expression levels in stem, leaf and panicle. To further confirm the high expression level of *OsOXO4* in panicles, we generated transgenic *Nipponbare* plants in which the expression of a β-glucuronidase (GUS) was driven by the promoter of *OsOXO4*. Strong GUS activity was detected in panicles at the booting stage and heading stage (Fig. 1C), agreeing well with the result from quantitative RT-PCR.

### Overexpression of *OsOXO2*, *OsOXO3* and *OsOXO4* Enhances Panicle Blast Resistance in Rice

**Fig. 2:**
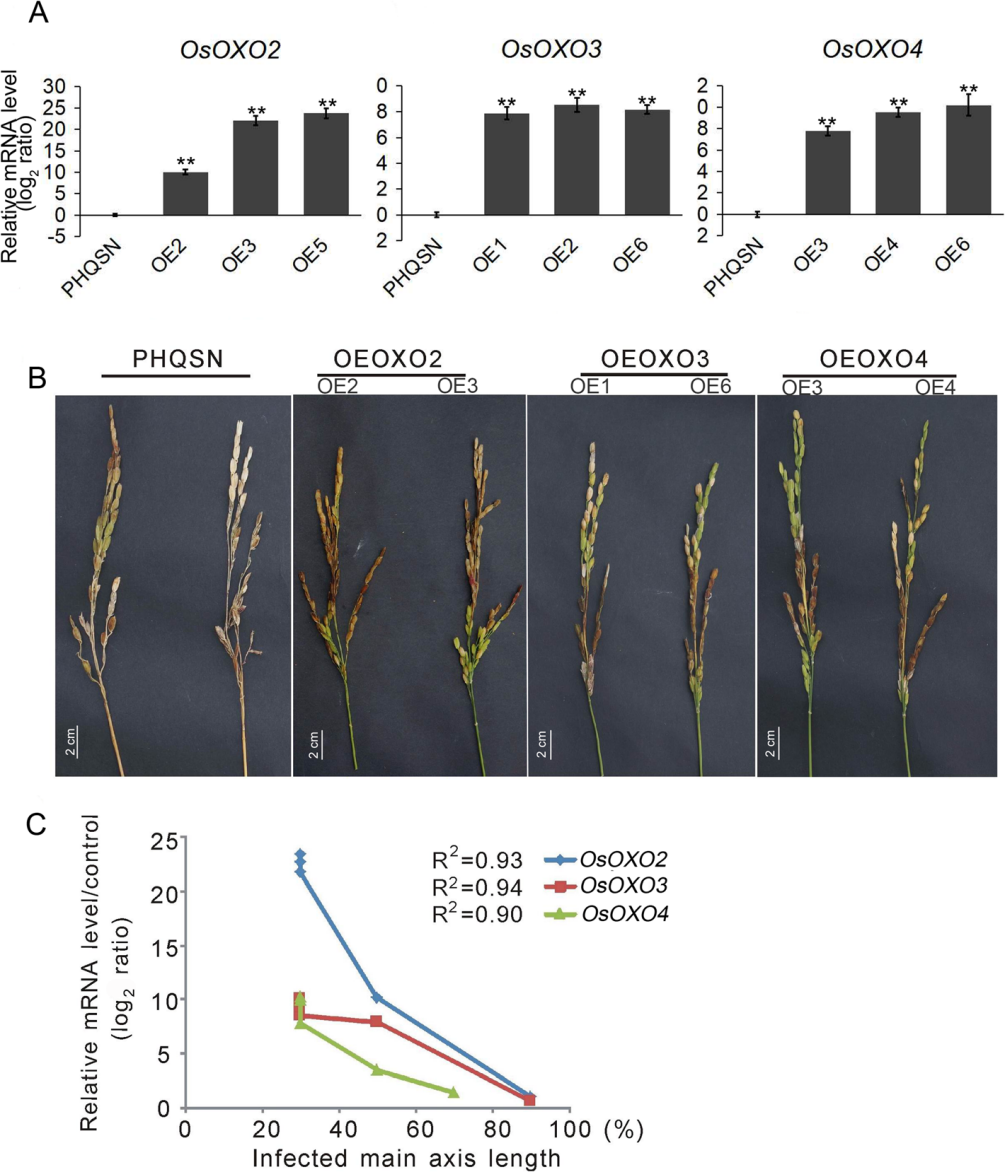
The disease phenotypes of the *OXO* gene overexpressing plants before and during panicle blast infection. OEOXO2 indicates the plants overexpressing *OsOXO2*, OEOXO3 indicates the plants overexpressing *OsOXO3* and OEOXO4 indicates the plants overexpressing *OsOXO4*. **a** Transcription analysis of *OsOXO2*, *OsOXO3* and *OsOXO4* in their corresponding overexpressing plants. Error bars indicate the SD from three biological replicates. ** indicates a statistically significant difference compared with PHQSN (*t* test, *P* < 0.01). **b** Disease phenotypes of OEOXO2, OEOXO3, OEOXO4, and the empty vector control (PHQSN) plants at the heading stage after inoculation with *M. oryzae* using the cotton-wrapping method. **c** Correlation analysis between enhanced resistance and the expression levels of *OsOXO2*, *OsOXO3* and *OsOXO4* in the overexpressing plants after panicle blast inoculation. The correlation coefficient is calculated by linear regression

To confirm the function of the three *OXO* genes in panicle blast resistance in rice, we generated the *OsOXO2*, *OsOXO3* and *OsOXO4* overexpressing plants in the japonica *Nipponbare* background, named OEOXO2, OEOXO3 and OEOXO4, respectively. The transgenic plants showed no obvious differences in non-target traits compared to *Nipponbare* plants and were fertile. Three independent homozygous lines of each *OXO* gene were used for panicle blast resistance evaluation. Quantitative RT-PCR analysis showed that the transcript levels of the three *OXO* genes were significantly increased in their corresponding transgenic lines (Fig. 2a). Cotton-wrapping inoculation with *M. oryzae* isolate GD08-T13 showed that the infected main axis length was 82.1% for *Nipponbare* plants and 83.2% for the transformed empty vector control (PHQSN) plants, but 31.46% to 35.25% for the *OXO* overexpressing plants (Table 1, Fig. 2b). The enhanced panicle blast resistance was correlated with the increased expression of *OXO* genes in all transgenic plants (Fig. 2c). However, there were no significant difference in diseased leaf area, lesion size and fungal biomass between the *OXO* overexpressing plants and the control plants (PHQSN) after blast inoculation (Table S2, Fig. S1).

**Table 1:** The infected main axis length of control (PHQSN) and *OXO* overexpressing plants after inoculation

Name^**a**^	Total number^**b**^	Infected main axis length (%)^**c**^	***P-value***^**d**^
PHQSN	12	83.231 ± 8.36	
Nip	7	82.1 ± 7.66	0.3924
OEOXO2(OE2)	15	35 ± 7.07	1.2362E-09
OEOXO2(OE3)	15	32.66 ± 5.62	1.13E-14
OEOXO2(OE5)	14	32.85 ± 5.78	5.77868E-14
OEOXO3(OE1)	12	31.46 ± 5.45	2.05E-14
OEOXO3(OE2)	13	34.15 ± 6.22	1.75E-14
OEOXO3(OE6)	13	33.46 ± 6.88	1.14298E-12
OEOXO4(OE3)	12	32.36 ± 5.78	1.85E-13
OEOXO4(OE4)	15	35.25 ± 5.32	2.03E-14
OEOXO4(OE6)	17	34.11 ± 6.90	1.62594E-14

^a^ *PHQSN* the transformed empty vector control plant, *Nip* Nipponbare; OEOXO2, OEOXO3, OEOXO4: the *OXO2, OXO3, OXO4* overexpressing plants; ^b^ the number of individual plants used for blast inoculation; ^c^ Infected main axis length (%) = the infected main axis length / the main axis length of the inoculated panicle × 100. Each value represents the mean ± standard error. ^d^ significant level of the difference by comparing with control PHQSN in *t*-test

### *OsOXO2*, *OsOXO3* and *OsOXO4* Silencing Rice Plants are More Susceptible to Panicle Blast

**Fig. 3:**
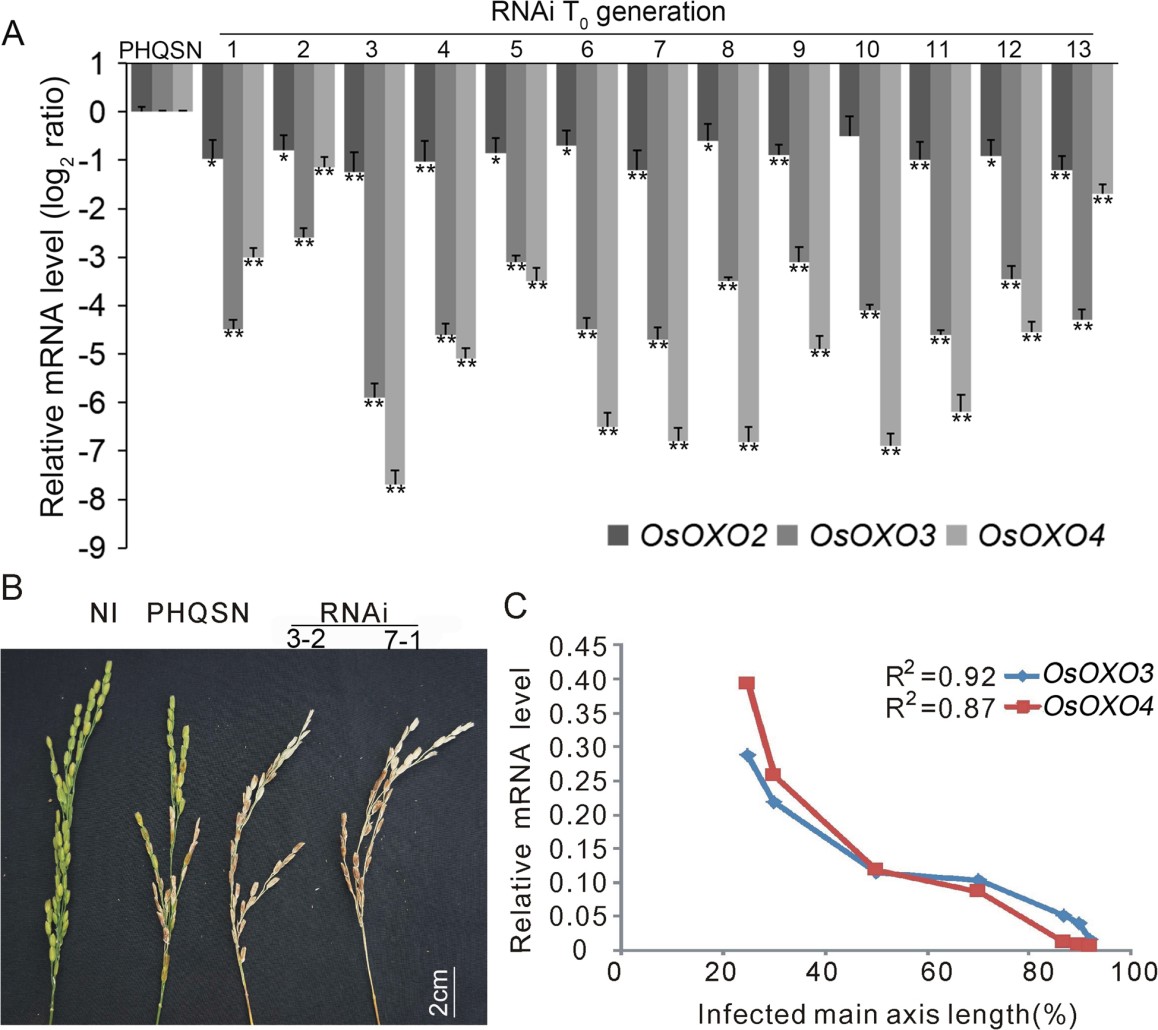
The disease phenotypes of *OXO* silencing (RNAi) plants after panicle blast infection. **a** Transcription analysis of *OsOXO2*, *OsOXO3* and *OsOXO4* in the RNAi plants by quantitative RT-PCR.Error bars indicate the standard deviation (SD) from three biological replicates and asterisks indicate statistically significant differences compared with ck (PHQSN) (*t* test, **P < 0.01, *P < 0.05). **b** Disease phenotypes of the RNAi and control (PHQSN) plants at the heading stage after inoculation with *M. oryzae* using the cotton-wrapping method. NI indicates the non-inoculation panicle. **c** Correlation analysis between decreased resistance and the expression levels of *OsOXO3* and *OsOXO4* in the RNAi plants after panicle blast inoculation. The correlation coefficient is calculated by linear regression

To further confirm the function of *OXO* genes in panicle blast resistance, we generated the *OXO* silencing plants in *Nipponbare* using an RNAi vector containing a 327 bp homologous coding sequence among *OsOXO2*, *OsOXO3* and *OsOXO4*. We obtained 13 silenced transgenic lines, which showed no differences in the non-target traits compared to the wild type *Nipponbare* and were fertile. Quantitative RT-PCR analysis showed that the expression levels of *OsOXO2*, *OsOXO3* and *OsOXO4* was remarkably decreased compared to the control plants (PHQSN) (Fig. 3a). The *OXO* silencing plants exhibited reduced resistance to panicle blast, with higher percentages of infected main axis length in both T_0_ and T_2_ generation plants when compared to the control plants (Table 2, Fig. 3b). The reduced expression levels of *OsOXO3* and *OsOXO4* were correlated with increased percentages of infected main axis length in the silencing plants (Fig. 3c). However, there were no significant difference in diseased leaf area, lesion size and fungal biomass between the *OXO* silencing plants and the control plants (PHQSN) after blast inoculation (Table S3, Fig. S1). The results from gene overexpressing and silencing experiments suggest that *OsOXO2*, *OsOXO3* and *OsOXO4* positively regulate panicle blast resistance in rice.

**Table 2:** The infected main axis length of control (PHQSN) and *OXO* silencing plants after inoculation

Name^**a**^	Total number^b^	Infected main axis length(%)^c^	***P-value***^**d**^
PHQSN	10	38.21 ± 2.73	
Nip	7	37.14 ± 4.18	0.412172464
RNAi (T_0_ generation)	13	72.5 ± 9.57	5.39314E-05
RNAi (3–2, T_2_ generation)	17	75.29 ± 10.27	1.23875E-07
RNAi (7–1, T_2_ generation)	11	73.63 ± 12.86	1.56343E-05

^a^ *PHQSN* the transformed empty vector control plant, *Nip* Nipponbare; RNAi (3–2) and RNAi (7–1) are *OXO* gene silenced lines in T_2_ generation; ^b^ the number of plants used for panicle blast inoculation; ^c^ Infected main axis length (%) = infected main axis length/main axis length of the inoculated panicle × 100. Each value represents the mean ± standard error; ^d^ significant level of the difference by comparing with control PHQSN in *t*-test

### *OsOXO2*, *OsOXO3* and *OsOXO4* Modulate the Expression of Defense-Related Genes

**Fig. 4:**
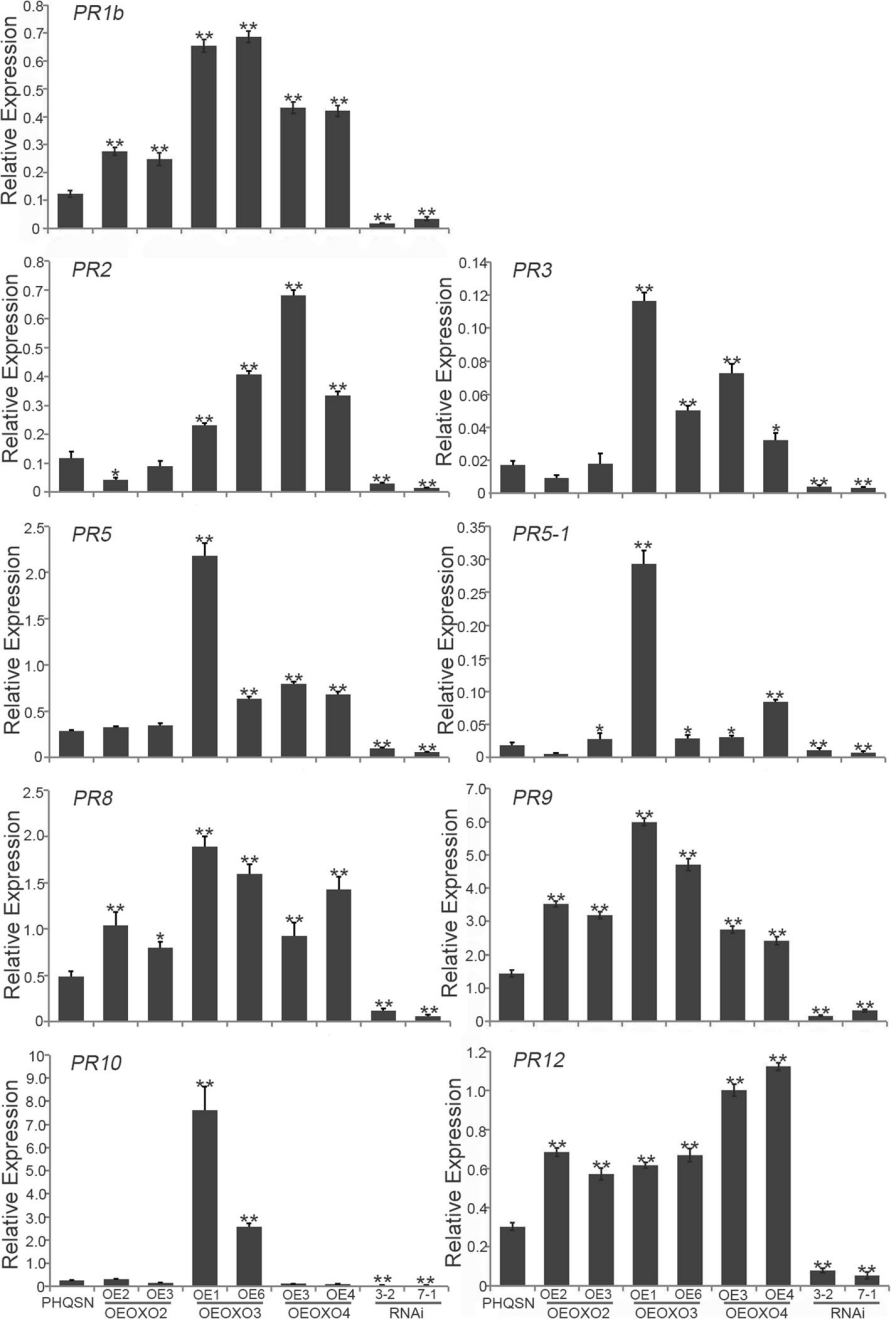
Expression analysis of pathogenesis-related (*PR*) genes in the *OXO* transgenic plants and control plants by quantitative RT-PCR. Error bars indicate the SD from three biological replicates and asterisks indicate statistically significant differences compared to the control plants (*t* test, **P < 0.01; *P < 0.05)

It is well-known that *PR* genes play important role in plant defense responses (Kaur et al. 2017). Previous study reported that the expression levels of *PR* genes were significantly increased in *OsERF83* overexpressing plants which showed enhanced rice blast resistance (Tezuka et al. 2019). In this study, the expression levels of several *PR* genes were analyzed in the *OXO* transgenic plants and control plants (PHQSN). The results showed that the expression levels of all the chosen *PR* genes were up-regulated in OEOXO3, and all the others except *PR10* were up-regulated in OEOXO4 plants, while only the transcriptions of *PR1b*, *PR8*, *PR9* and *PR12* were up-regulated in OEOXO2 compared to the control plants. Moreover, all the nine *PR* genes were down-regulated in *OXO* silencing plants compared to the control plants, suggesting that the three *OXO* genes may mediate different PR-involved disease resistance regulatory pathway in rice (Fig. 4).

### The *OsOXO2*, *OsOXO3* and *OsOXO4* Influence the Endogenous Levels of H_2_O_2_ in the Transgenic Plants

**Fig. 5:**
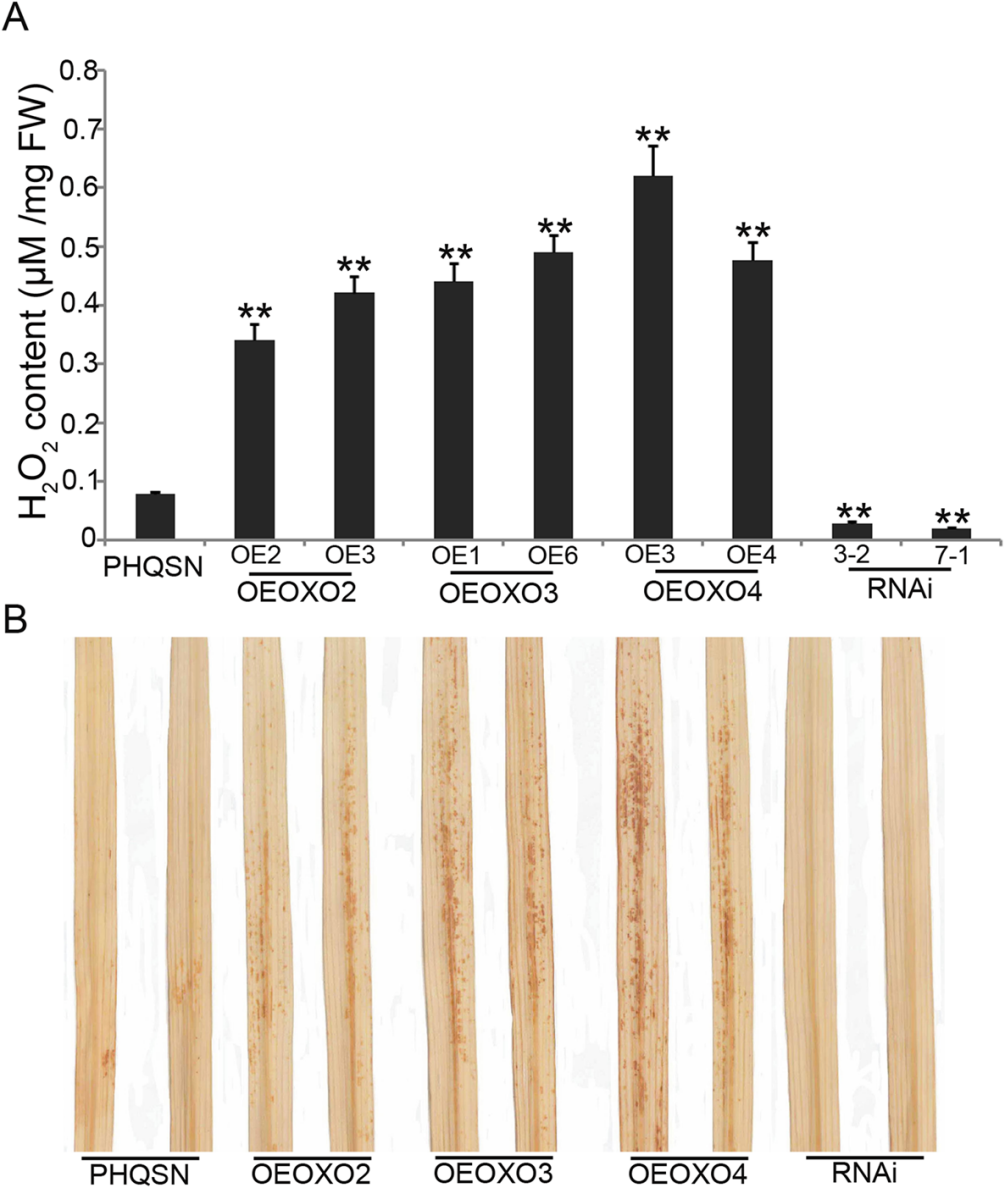
Hydrogen peroxide (H_2_O_2_) contents in the *OXO* transgenic plants and control plants. **a** H_2_O_2_ contents in the panicles of PHQSN, OEOXO2, OEOXO3, OEOXO4 and RNAi plants. **b** DAB staining of the leaves for PHQSN, OEOXO2, OEOXO3, OEOXO4 and RNAi plants. Error bars indicate the SD of at least ten biological replicates and ** indicates significant differences between the transgenic and control plants (*t* test, *P* < 0.01). FW means fresh weight

Previous studies have shown that higher levels of H_2_O_2_ contribute to *OXO* genes mediated fungal resistance in plants (Lamb and Dixon 1997; Carter et al. 1998; Wan et al. 2009). To confirm if the three *OXO* genes mediated panicle blast resistance is associated with the altered endogenous levels of H_2_O_2_, we firstly measured the H_2_O_2_ concentrations in the panicles of control (PHQSN) and transgenic plants using the xylenol orange method (Kim and Hwang 2014). The results showed that the H_2_O_2_ concentrations were significantly higher in the *OXO* overexpressing plants, but were lower in the *OXO* silencing plants when compared with the control plants (Fig. 5a). Similar results were also observed in leaves using the DAB staining method (Thordal-Christensen et al. 1997). Brown staining was observed in the leaves of both control and *OXO* overexpressing plants, but the staining was stronger in the *OXO* overexpressing plants than in control plants. No visible staining was observed in the leaves of *OXO* silencing plants (Fig. 5b).

### The Three *OXO* Genes Mediated Panicle Blast Resistance is Associated with Activation of the JA and ABA Signaling Pathways but Suppression of the SA Signaling Pathway

**Fig. 6:**
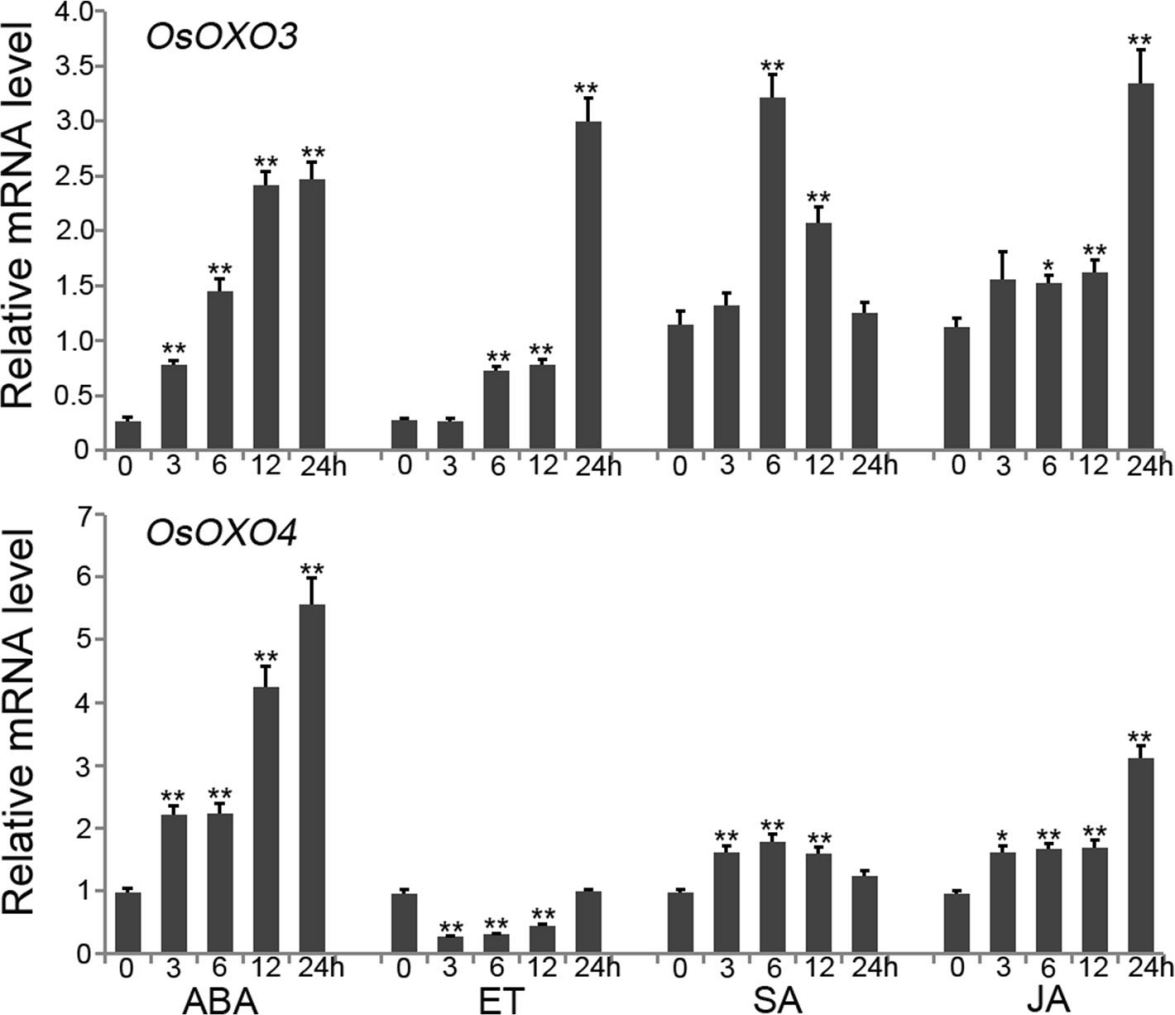
Time-course transcription analysis of *OsOXO3* and *OsOXO4* in leaves after abscisic acid (ABA), ethylene (ET), salicylic acid (SA), jasmonic acid (JA) treatments by quantitative RT-PCR. Error bars indicate the SD from three biological replicates and asterisks indicate statistically significant differences compared with water treatment (*t* test, **P < 0.01 and *P < 0.05)

To dissect the potential mechanisms of *OXO* genes-mediated panicle blast resistance in rice, we firstly analyzed the cis-elements in the promoters of *OsOXO2*, *OsOXO3* and *OsOXO4*, respectively. One ethylene (ET)-responsive element (ERE), two TC-rich repeats involved in defense and stress responses and one salicylic acid (SA)-responsive element (TCA-element) were found in the promoter of *OsOXO2*. Three abscisic acid (ABA)-responsive elements (ABREs), two methyl jasmonic acid (MeJA)-responsive elements (CGTCA-motif and TGACG-motif) and one TC-rich repeats were identified in the promoters of both *OsOXO3* and *OsOXO4* (Table S4). The existence of TC-rich repeats further supported the regulatory roles of *OsOXO2*, *OsOXO3* and *OsOXO4* in disease resistance. Meanwhile, the presence of the hormone response elements in the promoter region implies that the expression of the three *OXO* genes could be regulated by these hormones. To confirm this inference, we treated the wild-type *Nipponbare* plants with exogenous ABA, SA, ET and JA at the three leaf stage, respectively. The expression levels of the three *OXO* genes in leaf were analyzed both before and after hormone treatments using quantitative RT-PCR. Unfortunately, the expression of *OsOXO2* could not been detected. The expression of *OsOXO3* and *OsOXO4* were significantly induced by ABA, SA and JA treatment (Fig. 6). Transcription of *OsOXO3* was significantly induced while the transcription of *OsOXO4* was remarkably reduced by ET treatment (Fig. 6). These observations indicated that ABA, SA and JA may act upstream of *OsOXO3* and *OsOXO4* to induce their expression.

**Fig. 7:**
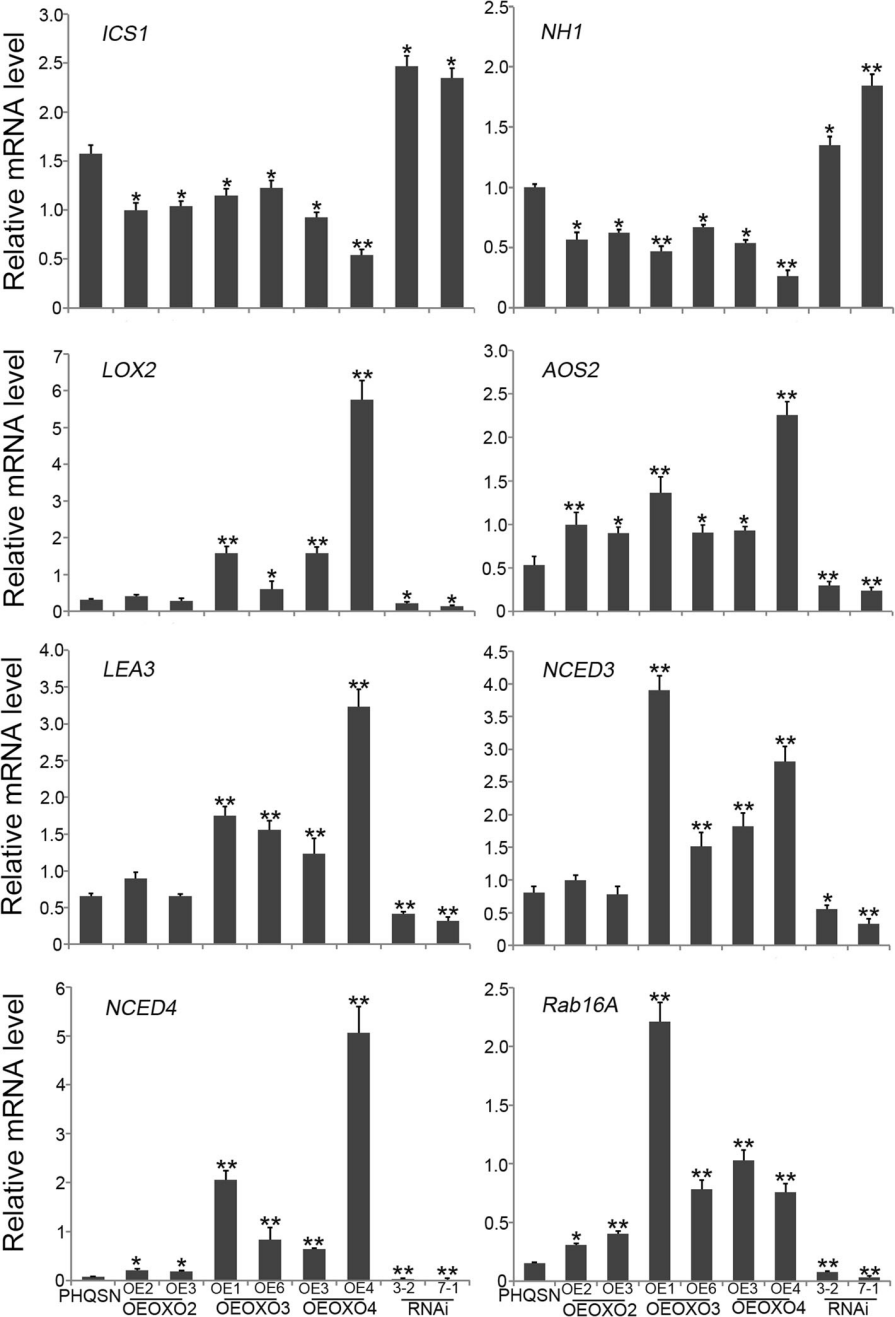
The expression of plant hormone related genes in panicles of the *OXO* transgenic plants and control plants by quantitative RT-PCR. Error bars indicate the SD from three biological replicates and asterisks indicate statistically significant differences compared to the control plants (*t* test, **P < 0.01 and *P < 0.05)

To further validate the relationship between the three *OXO* genes and hormone signaling pathways, we analyzed the expression patterns of several well-known stress-related genes that were involved in ABA, JA or SA signaling pathways in panicle in all the *OXO* transgenic lines, including *ICS1* and *NH1* which were related to the SA pathway (Deng et al. 2012), *LOX2* and *AOS2* which were related to the JA signaling pathway (Deng et al. 2012; Liu et al. 2016) and ABA signaling pathway related genes *LEA3*, *NCED3*, *NCED4* and *Rab16A* (Liu et al. 2013; Chen et al. 2015). Compared with the control plants, the transcription levels of *ICS1* and *NH1* were significantly decreased in OEOXO2, OEOXO3 and OEOXO4 plants. In contrast, the transcription level of *LOX2* was increased in OEOXO3 and OEOXO4 plants, while the transcription of *AOS2* was induced in all OEOXO2, OEOXO3 and OEOXO4 plants. The expression levels of *LEA3* and *NCED3* were significantly higher in OEOXO3 and OEOXO4 plants than in control plants, while the transcription levels of another two ABA-dependent pathway related genes *NCED4* and *Rab16A* were significantly higher in all OEOXO2, OEOXO3 and OEOXO4 plants than in control plants (Fig. 7). Just contrary to the results of overexpressing plants, the expression of *ICS1* and *NH1* were significantly up-regulated in the *OXO* silencing plants compared to the control plants, and the expression levels of *LOX2*, *AOS2*, *LEA3*, *NCED3*, *NCED4* and *Rab16A* were remarkably lower in the *OXO* silencing plants than in the control plants (Fig. 7). These results together suggest that the disease resistance conferred by *OsOXO2*, *OsOXO3* and *OsOXO4* could be associated with activation of JA and ABA signaling pathways while suppression of SA signaling pathway.

## Discussion

### *OsOXO2*, *OsOXO3* and *OsOXO4* Positively Regulate Panicle Blast Resistance in Rice

Since oxalate oxidases were first isolated and characterized in barley and wheat, they have been reported to play important roles in disease resistance in many plant species (Hu et al. 2003; Livingstone et al. 2005; Welch et al. 2007; Dong et al. 2008; Walz et al. 2008; Barman and Banerjee 2015; Yang et al. 2019). The four *OXO* genes in cluster on chromosome 3 have been reported to be co-localized with a QTL for rice blast resistance (Carrillo et al. 2009). However, the other study showed that the transgenic plants overexpressing *OsOXO1* or *OsOXO4* of Zhonghua11 did not show improved resistance to rice blast disease (Zhang et al. 2013). To further confirm the actual functions of the three *OXO* genes on blast resistance, we used BC10, a strong blast-resistant line (Liu et al. 2004) instead of Zhonghua11, a blast susceptible variety in the study conducted by Zhang et al. (2013) for cloning of the *OXO* genes in the present study. We identified that the expression of *OsOXO2*, *OsOXO3* and *OsOXO4* were induced by panicle blast infection in blast resistant line BC10 in microarray experiments and quantitative RT-PCR assays. All the overexpressing plants showed enhanced panicle blast resistance as manifested by the lower percent infected main axis length when compared with the control plants. In contrast, the silencing plants exhibited decreased panicle blast resistance with higher percent infected main axis length than the control plants. However, there were no significant difference in diseased leaf area between the transgenic plants (overexpressing and silencing) and the control plants (Table S2, Table S3 and Fig. S1). From these results, we can conclude that the three *OXO* genes (*OsOXO2*, *OsOXO3* and *OsOXO4*) function as positive regulators to modulate panicle blast resistance in rice. These results are different from the previous study (Zhang et al. 2013). We believed that the conflict could be attributed to the use of different materials for cloning of the three *OXO* genes. To confirm this inference, we compared the sequences of the protein coding regions of the three *OXO* genes between BC-10 and Zhonghua11 (Fig. S2). The results revealed that there were several SNPs which lead to the changes of translated proteins. The changes of translated proteins could result in difference in blast resistance. The fact that the three *OXO* genes have positive functions in regulating panicle blast resistance but no effect in leaf blast resistance provides a new evidence for the difference between leaf blast resistance and panicle blast resistance in rice.

### The Panicle Blast Resistance Conferred by *OsOXO2*, *OsOXO3* and *OsOXO4* is Associated with the Accumulation of H_2_O_2_ and the Expression of *PR* Genes

The enzymes encoded by *OXO* genes degrade oxalate acid to generate CO_2_ and H_2_O_2_, the latter has been proved to play a key role in plant disease resistance through cell wall modification by cross linking of plant cell wall proteins in papillae at the infection sites (Olson and Varner 1993; Wei et al. 1998; Liu et al. 2016; Xu et al. 2018; Li et al. 2019; Pei et al. 2019). In the present study, our results showed that the H_2_O_2_ concentrations in panicles were significantly higher in the *OXO* overexpressing plants, but were lower in the *OXO* silencing plants when compared with that in the control plants (Fig. 5a), suggesting that the panicle blast resistance conferred by *OsOXO2*, *OsOXO3* and *OsOXO4* was associated with the accumulation of H_2_O_2_. Interestingly, higher H_2_O_2_ concentrations were also observed in the leaves of the *OXO* overexpressing plants whereas lower H_2_O_2_ contents were also observed in the leaves of the *OXO* silencing plants compared to that in the control plants. However, there were no significantly differences in disease leaf area, lesion size and fungal biomass among the control, OE and RNAi plants (Table S2, Table S3 and Fig. S1). Different disease reactions of the *OXO* transgenic plants in panicle blast and leaf blast might indicate the different mechanisms between leaf blast resistance and panicle blast resistance in rice and the leaf blast resistance is not H_2_O_2_-dependent. There could be other mechanisms responsible for leaf blast resistance. The other possible explanation is that the H_2_O_2_ concentrations in the leaves of *OsOXO2*, *OsOXO3* and *OsOXO4* overexpression plants could be still not enough to resist against blast pathogen in leaves. Further study is needed to elucidate this issue.

The previous studies suggest that H_2_O_2_ functions as a secondary messenger to activate *PR* gene expression in plants (Hammond-Kosack et al. 1994; Carter et al. 1998). Here, we also identified that the expression levels of several *PR* genes were significantly up-regulated in *OXO* overexpressing plants (which harbors more H_2_O_2_) when compared to the control plants. This is consistent with the previous report that the sunflower plants overexpressing a wheat *OXO* gene could regulate a number of defense-related genes after pathogen infection (Hu et al. 2003). However, it should be noted that the expression patterns of the *PR* genes were different among different *OXO* gene overexpressing plants. For instance, the induction of *PR2, PR3*, *PR5* and *PR5–1* were identified in the OEOXO3 and OEOXO4 plants but not in the OEOXO2 plants. The induction of *PR10* was only observed in the OEOXO3 plants, and the induction of *PR1b*, *PR8*, *PR9* and *PR12* were identified in all the OEOXO2, OEOXO3 and OEOXO4 plants. These results implied that though *OsOXO2*, *OsOXO3* and *OsOXO4* all positively regulate panicle blast resistance in rice, their regulatory mechanisms may be different at least to some extent.

### The Important Roles of Hormone Signaling Pathways in *OsOXO2*, *OsOXO3* and *OsOXO4* Mediated Panicle Blast Resistance in Rice

Phytohormones are the well-known endogenous signal molecules that function in diverse biological processes including plant defense responses. Phytohormones generate and transmit distinct defense signals and the crosstalks between different hormones have been considered as universal defense responses employed by many plant species (Robert-Seilaniantz et al. 2011; Yang et al. 2013; Huot et al. 2014; Takatsuji and Jiang 2014; Yang et al. 2019). In this study, the crosstalks between JA, SA and ABA were identified for the panicle blast resistance conferred by *OsOXO2*, *OsOXO3* and *OsOXO4*. We discovered that there were JA, SA and ABA response elements in the promoter region of the three *OXO* genes, and the expression levels of *OsOXO3* and *OsOXO4* were significantly induced by exogenous JA, SA and ABA. Furthermore, our results showed that the transcription of *LOX2* and *AOS2* which are involved in JA biosynthesis, *LEA3*, *NCED3*, *NCED4* and *Rab16A* which are involved in the ABA signaling pathway were remarkably up-regulated in *OXO* gene overexpressing plants compared to the control plants. Reversely, the expression of *ICS1* and *NH1* which are involved in the SA signaling pathway were remarkably reduced in the overexpressing plants compared to the control plants. The expression patterns of these stress-related genes in the *OXO* silencing plants were just opposite to the results of the overexpressing plants. These results together suggest that the *OXO* genes mediated panicle blast resistance may be regulated by ABA, SA and JA, and associated with the activation of JA and ABA signaling pathways but suppression of SA signaling pathway.

In general, JA and SA are in most cases antagonized in regulating plant disease resistance (Robert-Seilaniantz et al. 2011). Our results here also indicated the antagonized roles between JA and SA in rice blast resistance. ABA has been well known for its regulatory roles in abiotic stress response and plant development. However, recently, more and more studies have discovered the important roles of ABA in regulating plant biotic stresses (Nambara and Marion-Poll 2005; Ton et al. 2009; Jiang et al. 2010; Jiang et al. 2013; Liu et al. 2018). For instance, ABA has been identified to positively regulate plant resistance to *Alternaria brassicicola* and *Plectospharella cucumerina* in Arabidopsis (Nambara and Marion-Poll 2005; Ton et al. 2009). Furthermore, our previous study also demonstrated that the transcription factor *ONAC066* mediated leaf blast resistance is involved in suppression of ABA signaling pathway, indicating the negative role of ABA in leaf blast disease (Liu et al. 2018). Nevertheless, in this study, we found that the panicle blast resistance conferred by *OXO* genes is associated with the activation of ABA signaling pathway, suggesting the positive role of ABA in panicle blast disease. The opposite regulatory roles of ABA may also partially explain the differential mechanisms between leaf blast resistance and panicle blast resistance.

## Conclusion

In conclusion, we have confirmed that *OsOXO2*, *OsOXO3* and *OsOXO4* from blast-resistant line BC10 positively regulate panicle blast resistance in rice in the present study. The *OXO* genes could modulate the accumulation of H_2_O_2_ and expression levels of *PR* genes in plants. Moreover, the *OXO* genes mediated panicle blast resistance may be regulated by ABA, SA and JA, and associated with the activation of JA and ABA signaling pathways but suppression of SA signaling pathway. However, there are still some issues to be elucidated in the future study. For example, are there any additive effects among the three *OXO* genes (*OsOXO2*, *OsOXO3* and *OsOXO4*). What are the common and different mechanisms of the three *OXO* genes in panicle blast resistance? Further studies are needed to address these issues.

## Methods

### Vector Construction and Rice Transformation

For overexpression vectors construction, the coding region sequences of *OsOXO2*, *OsOXO3* and *OsOXO4* were amplified from the blast-resistant line BC10 using the primers in Supplemental Table 1. The RNAi vector was generated by cloning a homologous sequence among *OsOXO2*, *OsOXO3* and *OsOXO4* using the primers in Supplemental Table 1. The resulting products were cloned into pEASY-T1 (TransGen) vector and verified by sequencing. The entry clones for overexpressing plants were then inserted into PHQSN (modified from pCAMBIA1390) which harbors a CaMV*35S* promoter. The clone for RNAi plants was constructed into pRNAi-Ubi, which was suitable for generation of hairpin-RNA constructs. For *P*_*OsOXO4*_*-GUS* construction, ~ 2.0-kb fragment was amplified from the upstream of *OsOXO4* in BC10 genomic DNA using specific primers (Table S1). Then the fragments were sub-cloned into pCAMBIA1381Z. All the positive plasmids and control vectors were electroporated into *Agrobacterium tumefaciens* EHA105 and then introduced into calli of the cultivar *Nipponbare* via Agrobacterium-mediated genetic transformation.

### Total RNA Extraction and Real-Time Quantification of mRNAs

The samples for total RNA extraction in the study of hormone treatments were collected from leaves, the other samples panicles for total RNA extraction were collected from panicles. Total RNA was extracted with Trizol reagent (Invitrogen) and purified with NucleoSpin RNA Clean-up (MACHEREYNAGEL) according to the manufacturers’ instructions. RNA quality and quantity were assessed by formaldehyde denaturing agarose gel electrophoresis and spectrophotometry (Nanodrop-1000), respectively. The purified total RNA was reverse-transcribed using the Primescript™ RT reagent kit (Takara) to generate cDNA and quantitative RT-PCR was carried out using SYBR ExTaq™ (Takara). *EF1a* gene was chosen as a reference gene. Gene expression was quantified by the comparative CT method. Experiments were performed in triplicate, and the results were presented by their means ± standard derivation (SD). *T* test was used for statistical analysis. Gene-specific primers used were listed in Table S5**.**

### GUS Staining Analysis

We analyzed GUS activity in transgenic panicles by histochemical staining with 5-bromo-4-chloro-3-indolyl-b-Dglucuronicacid (X-Gluc) as described previously (Liu et al. 2016a). Briefly, we incubated the transgenic panicles overnight at 37 °C in staining buffer (100 mM sodium phosphate [pH 7.0], 10 mM EDTA, 0.5 mM K_4_Fe(CN)_6_, 0.5 mm K_3_Fe(CN)_6_, 0.1% [v/v] Triton X-100 and 1 mM X-Gluc) and then decolorized in 100% ethanol before photographed.

### Sub-Cellular Localization Analysis

We amplified the protein coding region of *OsOXO2*, *OsOXO3* and *OsOXO4* from BC10 using the primers in Table S5 and cloned them into the pGY1-mcherry vector to generate the OXO-mcherry fusion proteins, respectively. The plasmids of OsOXO2-mcherry, OsOXO3-mcherry and OsOXO4-mcherry were extracted using UNlQ-50 Column Plasmid Max-Preps Kit (Sango). For the transient expression assay, 1 μg of every plasmid DNA was introduced into rice protoplasts. After 24 h incubation at 28 °C without light, the rice protoplasts were observed and photographed under a laser confocal microscopy (Zeiss LSM710, Germany).

### *Cis*-Elements Analysis of the Promoter

We downloaded approximately 1500 bp sequences upstream of *OsOXO2*, *OsOXO3*and *OsOXO4* from MSU Rice Genome Annotation Project (http://rice.plantbiology.msu.edu/), respectively. The sequences were scanned by PLACE (http://www.dna.affrc.go.jp/PLACE/) for cis-acting element analysis.

### Phytohormone Treatments

Hormone treatments were performed using the same method as our previous study (Liu et al. 2016b). Mature seeds of *Nipponbare* were soaked in distilled water for 2 days and pre-germinated for 2 days at 30 °C without light. Germinated seeds were placed in a salver for incubation in a growth chamber at 28 °C, 70% relative humidity and 12 h photoperiod. When the seedlings grown to three-leaf stage, they were sprinkled with different plant hormone solution and distilled water (control), respectively. The concentration of each hormone solution was 100 μM. Sampling for RNA extraction was conducted at 0 h before treatment and 3 h, 6 h, 12 h, 24 h after treatment. The experiments were repeated twice.

### Evaluation of Disease Resistance

We got T_0_ transgenic plants by vector construction and rice transformation. T_1_ and T_2_ segregating progeny germinated from T_0_ transgenic seeds were grown in soil in greenhouse. *M. oryzae* isolate GD08-T13 inoculum was used for blast resistance evaluation and was prepared as described by Beltenev et al. (2007). For leaf blast inoculation, 2 week seedings were inoculated by spraying with spore suspension of *M. oryzae* isolate GD08-T13 (1 × 10^6^ spores/ml). Inoculated plants were maintained in a growth chamber (25 °C, 16000 Lux, and 100% relative humidity) in the dark for 24 h; then, the growth chamber was set to a photoperiod of 16 h of light and 8 h of darkness at 25 °C and 100% relative humidity. Disease was assessed 5 days after inoculation by measuring the disease leaf area percent. Each treatment was repeated three times. We also used the punch method as described by Ding et al. (2012) for leaf blast evalution. For panicle blast inoculation, cotton-wrapping inoculation method was used as described by Liu et al. (2016b). Briefly, we wrapped the upper-middle part of a panicle by cotton in 1–2 days after heading, and injected 2 ml spore suspension of 1 × 10^6^ spores/ml of GD08-T13 into the cotton and then wrapped the cotton with foil. Each inoculated panicle was sprayed with water for 2–3 min every 2 h to maintain the humidity. Disease was assessed at 3 weeks after inoculation by calculating the percentage of infected main axis length (infected main axis length/main axis length of the inoculated panicle). Each treatment was repeated twice. *T-* test was used for test the significant level of difference.

### The 3, 3′-Diaminobenzidine (DAB) Staining and Measurement of H_2_O_2_

The DAB staining of H_2_O_2_ was conducted according to the previously reported method (Thordal-Christensen et al. 1997; Kim and Hwang 2014). DAB was dissolved by sterile ddH_2_O and reduced PH to 3.8 with HCl to get the 1 mg/ml DAB staining solution. Similar leaves of the transgenic and PHQSN plants were selected and immersed into the DAB staining solution immediately. After 3 h incubation in a growth chamber at 28 °C with relative humidity of 60% and light intensity of 50 μmol m^− 2^ s^− 1^, the leaves were decolorized in a water-bath at 80–90 °C for 1 h. Then, the decolorized leaves were photographed.

The measurement of H_2_O_2_ was conducted using the hydrogen peroxide assay kit (Beyotime Institute of Biotechnology, China) as described in our previous study (Liu et al. 2016). The similar panicles of the transgenic and PHQSN plants were collected and grinded with liquid nitrogen. The 200 mL lysis buffer solution was added to the 10 mg panicle dry powder and blended fully. The supernatant was collected by centrifuging at 12,000 *g* for 5 min. Then, the 50 μL of the supernatants and 100 μL of test solutions were transferred to the test-tubes immediately and the mixed solution was kept for 30 min at room temperature. H_2_O_2_ concentration was monitored by measuring the absorbance at 560 nm using a Thermo Scientific Multiskan Spectrum (Thermo, USA).

## Supplementary Information

**Additional file 1 : Figure S1.** The leaf disease phenotype and fungal biomass of the *OXO* over-expression and RNAi plants. A. the leaf blade state of CK (PHQSN) and transgenic plants at 5th day after inoculation by spraying with spore suspension. B. the disease area of CK (PHQSN) and transgenic plants at 10th day after inoculation by using the punch method. C. the fungal biomass of CK (PHQSN) and transgenic plants after inoculation by using the punch method.

**Additional file 2 : Figure S2.** Sequence alignments of *OsOXO2*, *OsOXO3* and *OsOXO4* between Zhonghua 11 and the blast-resistant line BC10. The red bases indicate the changed bases of the *OXO* genes in BC10 compared to Zhonghua 11.

**Additional file 3 : Table S1.** Microarray data of OXO genes after panicle blast inoculation. The value is log_2_ ratio.

**Additional file 4 : Table S2.** The diseased leaf area of control (PHQSN) and OXO overexpressing plants after inoculation.

**Additional file 5 : Table S3.** The diseased leaf area of control (PHQSN) and OXO silencing plants after inoculation.

**Additional file 6 : Table S4.** The cis-elements identified in the promoters (1500 bp upstream from the transcriptional starting site) of OXO genes.

**Additional file 7 : Table S5.** Primers used for vector construction and quantitative RT-PCR analysis.

## Data Availability

The datasets supporting the conclusions of this article are provided within the article and its additional files.

## Abbreviations

OXO: Oxalate oxidases
PR: Pathogenesis-related
JA: Jasmonic acid
ABA: Abscisic acid
ET: Ethylene
SA: Salicylic acid
GUS: β-glucuronidase
QTL: Quantitative trait loci
*ICS1*: *Isochorismate synthase 1*
*NH1*: *Nonexpressor of PR1*
*LOX2*: *Lipoxygenase 2*
*AOS2*: *Allene oxide synthase 2*
*LEA3*: *Late embryogenesis abundant protein gene* 3
*NCED3/4*: *9-cis-epoxycarotenoid dioxygenase 3/4*
*Rab16A*: *ABA-responsive rice gene*
