# Supplementary figures and images for: The OsOXO2, OsOXO3 and OsOXO4 Positively Regulate Panicle Blast Resistance in Rice
Source: Rice (N Y). 2021 Jun 5;14:51. doi: 10.1186/s12284-021-00494-9 (PMC8179873; doi:10.1186/s12284-021-00494-9)

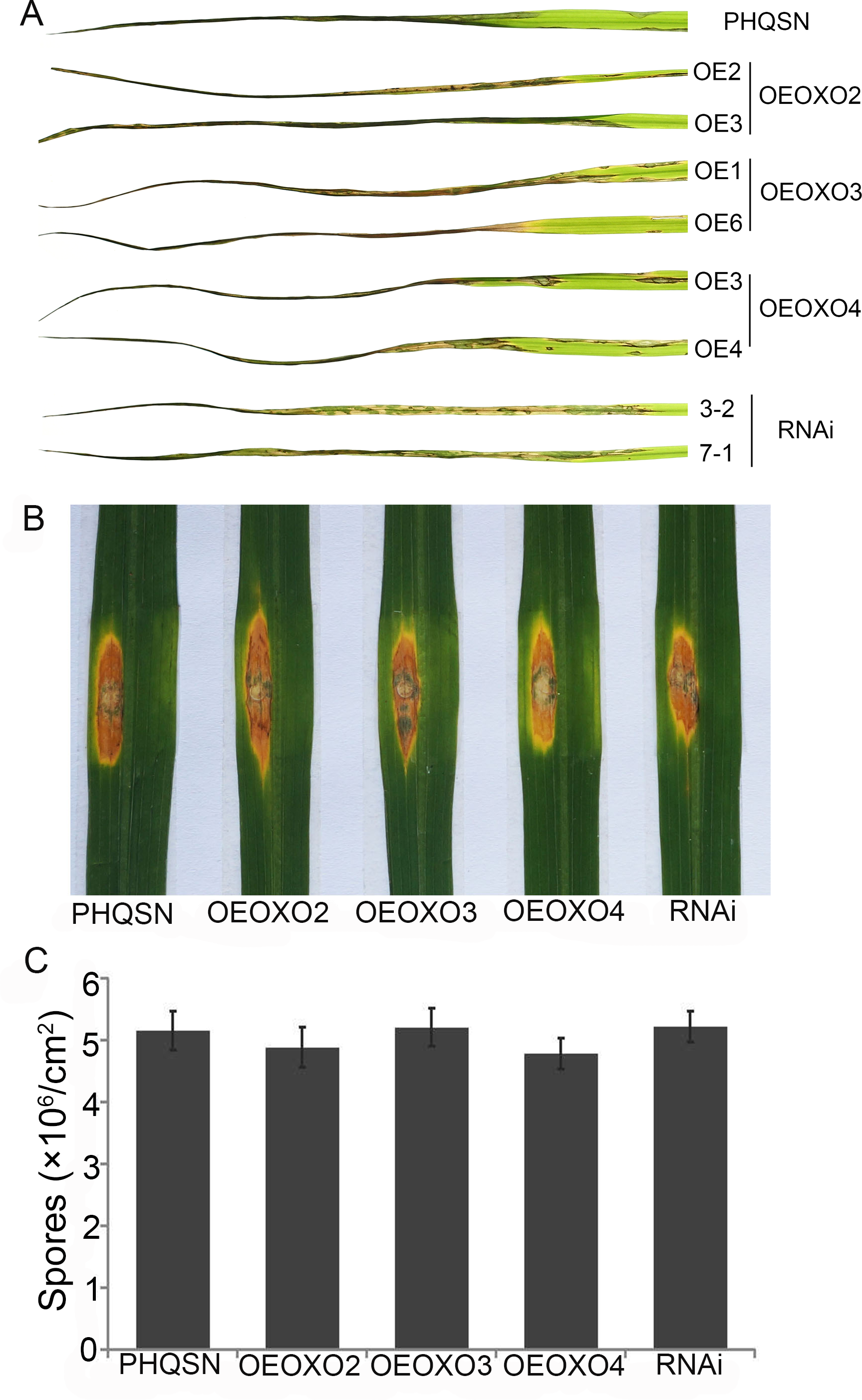

Supplement: Supplementary file 1 — Additional file 1 : Figure S1. The leaf disease phenotype and fungal biomass of the OXO over-expression and RNAi plants. A. the leaf blade state of CK (PHQSN) and transgenic plants at 5th day after inoculation by spraying with spore suspension. B. the disease area of CK (PHQSN) and transgenic plants at 10th day after inoculation by using the punch method. C. the fungal biomass of CK (PHQSN) and transgenic plants after inoculation by using the punch method. [file 12284_2021_494_MOESM1_ESM.tif]

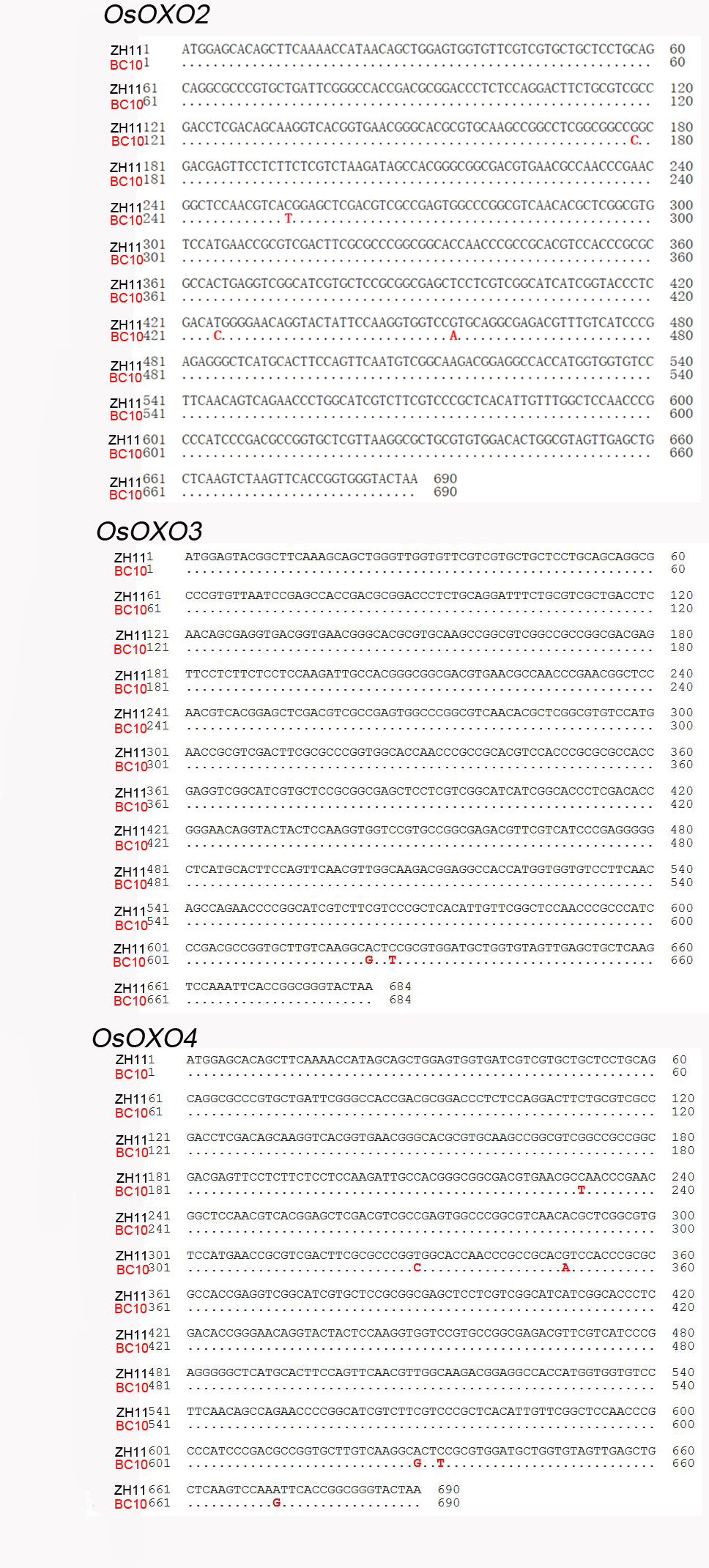

Supplement: Supplementary file 2 — Additional file 2 : Figure S2. Sequence alignments of OsOXO2, OsOXO3 and OsOXO4 between Zhonghua 11 and the blast-resistant line BC10. The red bases indicate the changed bases of the OXO genes in BC10 compared to Zhonghua 11. [file 12284_2021_494_MOESM2_ESM.tif]
