# Supplementary material for: The OsOXO2, OsOXO3 and OsOXO4 Positively Regulate Panicle Blast Resistance in Rice
Source: Rice (N Y). 2021 Jun 5;14:51. doi: 10.1186/s12284-021-00494-9 (PMC8179873; doi:10.1186/s12284-021-00494-9)
Supplement: Supplementary file 3 — Additional file 3 : Table S1. Microarray data of OXO genes after panicle blast inoculation. The value is log2 ratio. [file 12284_2021_494_MOESM3_ESM.docx]

| **gene** |  | **Panicle blast^a^** | |  |
| --- | --- | --- | --- | --- |
|  | **6h** | **12h** | **24h** | **48h** |
| Os03g0693800 (*OsOXO2*) | 3.64 | 5.97 | 4.38 | 5.24 |
| Os03g0693900 (*OsOXO3*) | 2.1 | 3.43 | 1.51 | 2.12 |
| Os03g0694000 (*OsOXO4*) | 3.31 | 5.43 | 4.99 | 4.35 |

**Table S1. Microarray data of *OXO* genes after inoculation. The value is log_2_ ratio.**

^a^ indicates the expression of the genes at different time after inoculation.
