# Supplementary material for: The OsOXO2, OsOXO3 and OsOXO4 Positively Regulate Panicle Blast Resistance in Rice
Source: Rice (N Y). 2021 Jun 5;14:51. doi: 10.1186/s12284-021-00494-9 (PMC8179873; doi:10.1186/s12284-021-00494-9)
Supplement: Supplementary file 4 — Additional file 4 : Table S2. The diseased leaf area of control (PHQSN) and OXO overexpressing plants after inoculation. [file 12284_2021_494_MOESM4_ESM.docx]

| **Name^a^** | **Total number^b^** | **Diseased leaf area (%)^c^** | ***P-value*^d^** |
| --- | --- | --- | --- |
| PHQSN | 17 | 77.35±10.84 |  |
| Nip | 14 | 76.14±11.85 | 0.384497703 |
| OEOXO2(OE2) | 11 | 72.90±15.33 | 0.188228726 |
| OEOXO2(OE3) | 15 | 71.6±14.33 | 0.103520681 |
| OEOXO3(OE1) | 18 | 76.83±13.16 | 0.449859584 |
| OEOXO3(OE6) | 14 | 76.85±12.47 | 0.453292485 |
| OEOXO4(OE3) | 14 | 71.07±14.70 | 0.090921624 |
| OEOXO4(OE4)  ^a^ PHQSN: the transformed empty vector control plant; Nip: Nipponbare; OEOXO2, OEOXO3, OEOXO4: the *OXO2, OXO3, OXO4* overexpressing plants;**^b^** indicates the sum number of plants used for leaf blast resistance evalution; **^c^** indicates the proportion of disease leaf area in the total leaf area. Diseased leaf area (%)= infected leaf area/total leaf area×100. Each value represents the mean± standard error ; **^d^** is calculated by *t*-test using PHQSN as control. | 17 | 71.70±14.88 | 0.10760623 |

**Table S2. The diseased leaf area of control (PHQSN) and *OXO* over-expressing plants after inoculation with leaf blast.**
