# Supplementary material for: The OsOXO2, OsOXO3 and OsOXO4 Positively Regulate Panicle Blast Resistance in Rice
Source: Rice (N Y). 2021 Jun 5;14:51. doi: 10.1186/s12284-021-00494-9 (PMC8179873; doi:10.1186/s12284-021-00494-9)
Supplement: Supplementary file 5 — Additional file 5 : Table S3. The diseased leaf area of control (PHQSN) and OXO silencing plants after inoculation. [file 12284_2021_494_MOESM5_ESM.docx]

| **Name^a^** | **Total number^b^** | **Diseased leaf area (%)^c^** | ***P-value*^d^** |
| --- | --- | --- | --- |
| PHQSN | 17 | 77.35±10.84 |  |
| Nip | 14 | 76.14±11.85 | 0.384497703 |
| RNAi (3-2, T_2_ generation) | 10 | 78.28±8.95 | 0.4215049 |
| RNAi (7-1, T_2_ generation)  ^a^ PHQSN: the transformed empty vector control plant; Nip: Nipponbare; RNAi (3-2) and RNAi (7-1) are *OXO* gene silenced lines in T_2_ generation; **^b^** indicates the sum number of plants used for leaf blast resistance evalution; **^c^** indicates the proportion of disease leaf area in the total leaf area. Diseased leaf area (%)= infected leaf area/total leaf area×100. Each value represents the mean± standard error ; **^d^** is calculated by *t*-test using PHQSN as control. | 11 | 82±8.78 | 0.140154155 |

**Table S3. The diseased leaf area of control (PHQSN) and *OXO* silencing plants after inoculation with leaf blast.**
