# Supplementary material for: The OsOXO2, OsOXO3 and OsOXO4 Positively Regulate Panicle Blast Resistance in Rice
Source: Rice (N Y). 2021 Jun 5;14:51. doi: 10.1186/s12284-021-00494-9 (PMC8179873; doi:10.1186/s12284-021-00494-9)
Supplement: Supplementary file 6 — Additional file 6 : Table S4. The cis-elements identified in the promoters (1500 bp upstream from the transcriptional starting site) of OXO genes. [file 12284_2021_494_MOESM6_ESM.docx]

| **Cis-elements** | **Sequence** | ***OsOXO2*** | | ***OsOXO3*** | | ***OsOXO4*** | | **Function** |
| --- | --- | --- | --- | --- | --- | --- | --- | --- |
|  |  | **Number** | **Position(-)^*^** | **Number** | **Position^*^** | **Number** | **Position^*^** |  |
| ABRE | CACGTG |  |  | 3 | 188; 190; 197 | 3 | 265; 534; 630 | cis-acting element involved in the abscisic acid responsiveness |
| CGTCA-motif | CGTCA |  |  | 1 | 58 | 1 | 1075 | cis-acting regulatory element involved in the MeJA-responsiveness |
| ERE | ATTTCAAA | 1 | 742 |  |  |  |  | ethylene-responsive element |
| TC-rich repeats | ATTTTCTCCA | 2 | 447; 935 | 1 | 630 | 1 | 482 | cis-acting element involved in defense and stress responsiveness |
| TCA-element | GAGAAGAATA | 1 | 448 |  |  |  |  | cis-acting element involved in salicylic acid responsiveness |
| TGACG-motif | TGACG |  |  | 1 | 8 | 1 | 1075 | cis-acting regulatory element involved in the MeJA-responsiveness |

**Table S4. The cis-elements identified in the promoters (1500bp upstream from the transcriptional starting site) of *OXO* genes.**

**^*^**indicates the position in the upstream of the ORF region.
