# Supplementary material for: The OsOXO2, OsOXO3 and OsOXO4 Positively Regulate Panicle Blast Resistance in Rice
Source: Rice (N Y). 2021 Jun 5;14:51. doi: 10.1186/s12284-021-00494-9 (PMC8179873; doi:10.1186/s12284-021-00494-9)
Supplement: Supplementary file 7 — Additional file 7 : Table S5. Primers used for vector construction and quantitative RT-PCR analysis. [file 12284_2021_494_MOESM7_ESM.docx]

**Table S5. Primers used for vector construction and quantitative real-time PCR analysis.**

|  | forward primer (5'-3') | reverse primer (5'-3') |
| --- | --- | --- |
| OsOXO2-mcherry-F/R | ATGGAGCACAGCTTCAAAACCATAA | TAACGAGCACCGGCGTCGG |
| OsOXO3-mcherry-F/R | ATGGAGTACGGCTTCAAAGCA | GTACCCGCCGGTGAATTTGGA |
| OsOXO4-mcherry-F/R | ATGGAGCACAGCTTCAAAACCA | GTACCCGCCGGTGAATTTGGA |
| OsOXO4-GUS-F/R | ATTATTCAGGGATTCACACCGC | GGATGAACTAGCATTTGAGCCCTT |
| OsOXO2-OE-F/R | CGTCTCCTCACTGCTACTGTCA | CTCAACTACGCCAGTGTCCACA |
| OsOXO3-OE-F/R | ATGGAGTACGGCTTCAAAGCA | TTAGTACCCGCCGGTGAATTTG |
| OsOXO4-OE-F/R | TGTCACTGCGCTTCTTTCCTAG | ACACCATGAAAATGCTCTCGGA |
| RNAi-F/R | CTTCTCCTCCAAGATTGCCACG | GTCTTGCCGACATTGAACTGGA |
| *OsOXO2*-q-F/R | CGTCCTTCAACAGTCAGAACCCT | CTCAACTACGCCAGTGTCCACA |
| *OsOXO3*-q-F/R | ATGGAGTACGGCTTCAAAGCA | TGAGGTCAGCGACGCAGAAAT |
| *OsOXO4*-q-F/R | CGCGCTTCTTTCCTAGTTTCCTT | CTGAAGCTGTGCTCCATTGTTG |
| *PR1b*-q-F/R | CAAAACTCCCCGCAGGACTA | GAGGTTCTCGCCAAGGTTGT |
| *PR2*-q-F/R | CCTTCACCAAGTATCTGCGA | GTCTAGCGCATTCTGCAAAC |
| *PR3*-q-F/R | GGGTCGGCTACTACAAGAGG | TCCCTGCAGGCTATGTTATCT |
| *PR5*-q-F/R | CTGCAGGGACAGCAGATG | ACTTGGTAGTTGCTGTTTCCC |
| *PR5-1*-q-F/R | CACCTGCAGGGACAGCC | ATGGGCAGAAGACGACTTGG |
| *PR8*-q-F/R | TCTACGACGTGCAGAACAACTTCAG | TCCAACTCAACCACTGTGCAAGTAA |
| PR9-q-F/R | ACCAACATCGATTCCGCCTT | CTTGTTGGACAGCAGGTTGC |
| *PR10*-q-F/R | CCCTGCCGAATACGCCTAA | CTCAAACGCCACGAGAATTTG |
| PR12-q-F/R | CCACAGGTTCAAGGGCATGT | CTTCTTGCAGAAGCACTTGCG |
| *ICS1*-q-F/R | TATGGTGCTATCCGCTTCGAT | CGAGAACCGAGCTCTCTTCAA |
| *NH1*-q-F/R | CACGCCTAAGCCTCGGATTA | TCAGTGAGCAGCATCCTGACTAG |
| *LOX2*-q-F/R | GCATCCCCAACAGCACATC | AATAAAGATTTGGGAGTGACATATTGG |
| *AOS2*-q-F/R | CAATACGTGTACTGGTCGAATGG | AAGGTGTCGTACCGGAGGAA |
| *LEA3*-q-F/R | CGGCAGCGTCCTCCAAC | CGGTCATCCCCAGCGTG |
| *NCED3*-q-F/R | ACGTGATCAAGAAGCCGTACCT | GCTGGTCGAGCGGGATCT |
| *NCED4*-q-F/R | GCCGAGACACGCATTGG | GTGAAGGTGGCGACAGCAA |
| *Rab16A*-q-F/R | CACACCACAGCAAGAGCTAAGTG | TGGTGCTCC ATCCTGCTTAAG |
| *EF1α*-q-F/R | TTTCACTCTTGGTGTGAAGCAGAT | GACTTCCTTCACGATTTCATCGTAA |
